# Corkscrew Technique for Extraction of Premolars and Molars in Standing Sedated Horses: Cadaveric Study and Clinical Cases

**DOI:** 10.3390/ani14101439

**Published:** 2024-05-11

**Authors:** Joao D. Ferreira, José L. Méndez-Angulo

**Affiliations:** Méndez Hospital Equino, 14100 Córdoba, Spain; mendezhospitalequino@gmail.com

**Keywords:** premolar, molar, tooth, extraction, horse, head, buccotomy, corkscrew

## Abstract

**Simple Summary:**

Cheek teeth extractions are a laborious procedure. A minimally invasive transbucal screw extraction technique has been described. This technique requires several mallet strikes to disrupt the periodontal ligament to loosen the affected tooth. The objective of this study is to provide evidence that the use of a corkscrew-like mechanism is effective in completing cheek tooth extraction in standing sedated horses without the use of a mallet. The instruments and methodology described here can help equine practitioners to improve dental screw extractions. Further studies are needed to refine the instruments and procedures as well as evaluate both intraoperative and postoperative complications.

**Abstract:**

Several tooth extraction techniques are described in equine literature, and oral extraction techniques in standing sedated horses are popular among equine practitioners. The objectives of this study were to develop the corkscrew technique for cheek tooth extraction (CSET) in equine cadaver heads and evaluate this technique in clinical cases. We hypothesized that the CSET could be performed safely to extract cheek teeth in standing sedated horses. First, the CSET was attempted and developed in eight equine cadaver heads. Second, the CSET was performed in clinical cases between 2016 and 2020, and the following information was recorded: diagnosis, affected tooth, procedure duration, intraoperative difficulties, tooth size, postoperative complications, medication, hospitalization time, and 1-year follow-up. Sixteen CSET procedures were performed in eight equine skulls with a 75% success rate. In 24 clinical cases, 25 CSET procedures were attempted to extract 22 superior and 3 inferior cheek teeth. CSET was successful in 76% of procedures. Fractures of the tooth and stripping of screw threads were the major complications that led to the failure of CSET. CSET is a viable and safe technique to extract cheek teeth in standing sedated horses. Longitudinal drilling is a must for this technique to be successful.

## 1. Introduction

Exodontia of premolars and molars is the most used method to treat teeth disorders in horses [1,2]. Primary apical infection or apical infection secondary to periodontal disease due to dental trauma, diastemata, displaced or supernumerary teeth, crown fractures involving the pulp cavity, and infundibular caries are the major indications for the removal of cheek teeth [3,4]. Other indications for exodontia include retained deciduous premolars, wear abnormalities, and dental neoplasia [5,6]. Oral extraction techniques under standing sedation have become more popular as better sedation agents have become available [7]. Although it can be a physically hard and time-consuming process, tooth extraction has high success and very low complication rates [2,4,5,6,8]. Oral extraction is particularly challenging for teeth with long reserve crowns and intact periodontal ligaments or when there is a lack of clinical crown to be grasped [1].

When oral extraction fails or the dental conditions that are seen in the pre-extraction imaging diagnostics are not suitable for this type of procedure, surgical methods must be used. Repulsion and alveolar osteotomy have been proposed as surgical procedures for cheek teeth exodontia [1,9]. Both techniques can be safely performed under standing sedation or general anesthesia [9,10,11]. Repulsion is a well-accepted procedure among surgeons, but many important complications have been reported with this technique, such as alveolar sequestrum, draining tracts, oromaxillary fistulas, damage to adjacent teeth, and sinusitis [8,10,12]. Exposure of the tooth crown via alveolar osteotomy through a lateral buccotomy access is technically more demanding as the surgeon must work in proximity of vital structures, like facial vessels and nerves. This surgical technique also has a higher complication rate compared to oral extraction methods [4,8,11,12].

More recently, a minimally invasive buccotomy approach has been reported to extract cheek teeth with the use of an intradental screw [13]. For this technique, the mandible is laterally displaced with the help of a Günther speculum, and a small buccotomy is performed to drill a hole through the occlusal surface of the tooth. Then, a screw is placed in the tooth through the buccotomy, and a slotted mallet is used to disrupt the periodontal ligament and extract the tooth orally [13]. This technique requires a preliminary preparation of the tooth by breaking off the periodontal ligament with elevators and spaces passed through the trocar placed through the buccotomy. Although being a much more laborious technique, it permits oral exodontia even in teeth that lack clinical crowns, with similar success to the oral method and with fewer complications than other surgical techniques [14]. This technique works very well with short teeth, but it becomes harder with larger teeth because the hammering must be more aggressive to break the periodontal ligament, and it can end in the fracturing of the tooth. Additionally, some horses do not tolerate well the use of the mallet on their heads [14,15]. For these reasons and to avoid direct hammering on the head of the horse, we propose a modification of the screw extraction mechanism in this study. Instead of using a mallet to apply the extracting force, we have developed a corkscrew-like mechanism that is placed on the cheek teeth adjacent to the targeted tooth, which can subsequently exert a powerful and even extraction force to extract the affected tooth. Therefore, the objectives of the present study were (1) to design and attempt the corkscrew technique for dental extraction in equine cadaver heads and (2) to report our experience with this technique in clinical cases. Our hypothesis was that the corkscrew extraction technique (CSET) could be performed safely to extract cheek teeth in standing sedated horses.

## 2. Materials and Methods

### 2.1. Cadaver Model 

A cadaver model was used to develop the CSET. Eight equine skulls from horses ranging from 4 to 10 years old were collected from a slaughterhouse and kept refrigerated at 4 °C with the mouth open for seven days. The experiment with the cadavers was performed on two consecutive days, four heads per day. First, each head was placed in an anatomical position and firmly fixed to a wood block with duct tape. A Günther speculum was fitted in the mouth and opened, and the mandible was lateralized. Two cheek teeth were randomly chosen to be extracted from each head. The Triadan 11s were excluded due to their reserve crown configuration and location. 

**Figure 1:**
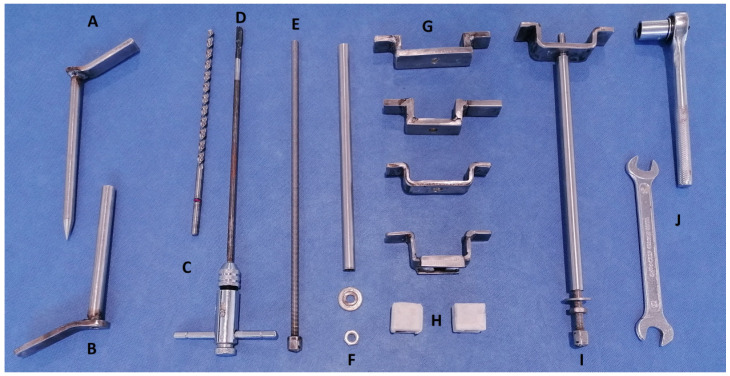
Photograph of the instruments designed for the corkscrew extraction technique (CSET). A—obturator, B—drill guide, C—drill bit, D—tap, E—screw, F—cylinder, washer, and nut, G—different sized bridges, H—plastic height adjusters, I—mounted “corkscrew” device, J—13 mm wrench and ratchet with 13 mm socket.

**Figure 2:**
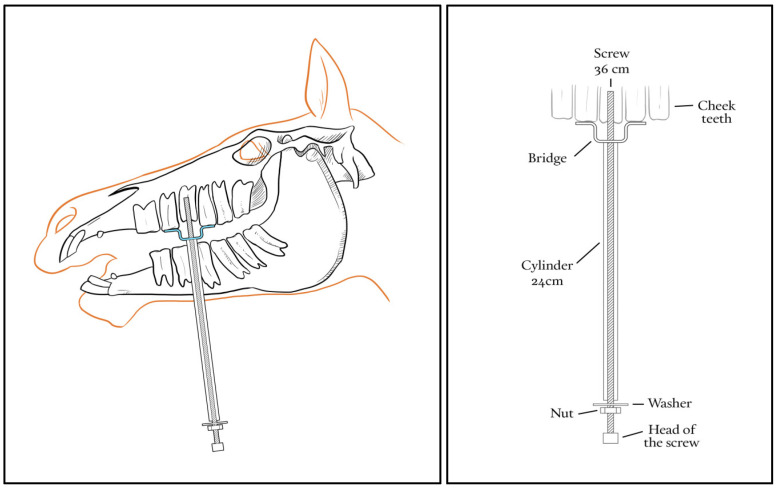
Diagrams showing the different parts of the CSTE device and how it must be mounted in the mount of the horse to extract a tooth.

All instruments required to perform the CSET were designed and manufactured in stainless steel with the help of a specialized blacksmith (Juan Muñoz-Perez, Córdoba, Spain). This kit included a drill, a 6.75 × 220 mm drill bit, a 130 mm drill guide (12 mm outside diameter × 1 mm thickness), an M8—1.25 × 245 mm tap, a 160 mm obturator (10 mm diameter), a 240 mm long cylinder (10 mm outside diameter × 1 mm thickness), an M8—1.25 × 320 mm screw with an M8 nut welded at one end, several bridges (90–120 mm length × 20–30 mm height made with 20 mm width × 5 mm thickness plate), M8 nuts, M8 × 16 mm washers, a 13 mm wrench, and a ratchet with a 13 mm socket (Figure 1). A diagram showing how the CSTE device is mounted in the mouth of a horse to extract a tooth is shown in Figure 2.

**Figure 3:**
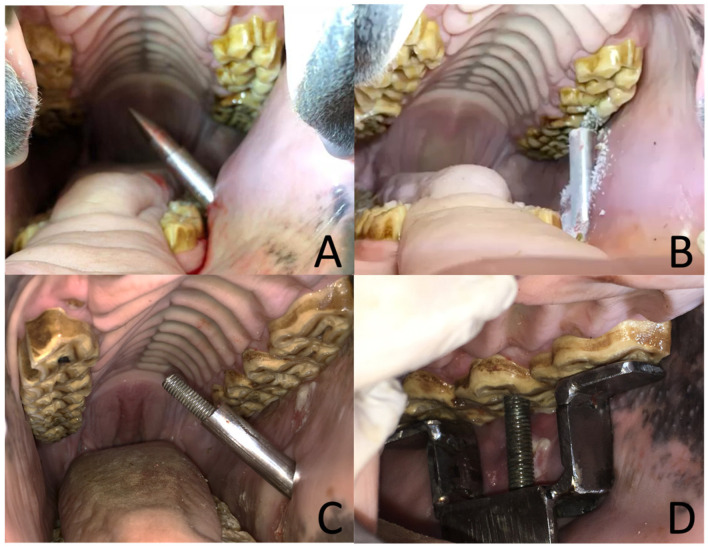
Sequence of images of the CSET. Image (**A**)—the obturator and the drill guide have been inserted through the buccotomy. Image (**B**)—a drill bit has been placed inside the drill guide, and tooth 207 is being drilled. Image (**C**)—the screw inside of the cylinder has been passed through the buccotomy. Image (**D**)—the screw has been passed through the hole in the bridge and threaded in the tooth hole. The CSET device is ready to attempt the extraction now.

For extraction of superior check teeth, the mandible was lateralized to the opposite site, and the buccotomy site was planned via oral palpation to be longitudinal to the targeted tooth and as ventral as possible but allowing at least 2 cm of separation to the gingiva of the mandible. For inferior check teeth, the mandible was lateralized to the same site in which the tooth was going to be extracted, and the buccotomy was planned similarly as for superior teeth but 2 cm from the maxillary gingiva. In both cases, a 1 cm long skin incision was made with a number 22 scalpel, and the drill guide was introduced through the cheek musculature with the help of the obturator and applying a moderate amount of pressure (Figure 3A). A hand was placed in the oral cavity to control this procedure and to protect the oral structures. The drill guide was advanced through the buccotomy until it contacted the center of the occlusal surface of the selected tooth, and the obturator was removed. Then, a drill bit was inserted in the drill guide, and a hole was drilled at the center of the occlusal surface of the tooth, trying to be as longitudinal as possible to the tooth in an apical direction (Figure 3B). Radiographs were taken during the drilling process to ensure the correct direction of the hole. Drilling was performed as deep as possible, but care was taken not to perforate the most apical aspect of the tooth. The hole was flushed with a spinal needle through the buccotomy, and treads were cut with the tap. Finally, the drill guide was removed from the cheek, and the cylinder with the screw inside was introduced through the buccotomy (Figure 3C). The bridge was placed over the targeted tooth orally, and the screw was inserted through the hole in the bridge and threaded into the tooth hole (Figure 3D). Care was taken so that the bridge sat well on the rostral and caudal teeth, and the cylinder was placed perpendicularly to the bridge. Once the corkscrew system was in place, the nut was tightened slowly against the cylinder, which pulled the tooth out of the alveolus gradually until the periodontal attachments were disrupted. During this procedure, the head of the screw was held in place with a ratchet. The targeted tooth was extracted about 1–2 cm from the alveolus, and at that point, the screw system was removed from the mouth, and the extraction was completed orally, either by hand or with forceps. Once the procedure was completed, the oral cavity was visually inspected to detect any possible damage caused to the alveolus, other teeth, or soft tissue structures. Complications during each procedure were recorded.

### 2.2. Retrospective Clinical Report

After the experience with cadaver heads and once the surgical team felt comfortable with the technique, clinical cases were recruited. Similar to the ex-vivo portion of the study, we excluded cases involving Triadan 11s. Only horses with intact affected teeth were included in this study. Clinical cases were restrained in stocks, a 14 G × 52 mm short intravenous (IV) jugular catheter was placed, and sedation was achieved with 0.01 mg/kg IV bolus of detomidine hydrochloride (Echuphar Veterinaria SLU, Barcelona, Spain) and re-dosed (0.005 mg/kg IV) as needed. Perioperative analgesia was provided by 2.2 mg/kg IV bolus of phenylbutazone (Zoetis Spain, S.L, Madrid, Spain). Perioperative antibiotics were administered at the surgeon’s discretion. Either a mandibular or a maxillary nerve block was performed with 20 mL of 2% lidocaine (B.Braun medical SA, Rubi, Barcelona, Spain). The mouth was rinsed with water before the placement of the mouth speculum. The horse’s head was placed on a headstand. Before the buccotomy, caution was taken to identify the parotid duct, facial vein, artery, and nerve. The procedure was performed as described previously. Once the tooth was extracted, the alveolus was washed with water and inspected by hand or oral endoscopy for dental fragments or any iatrogenic damage. Curettage of the alveolus through the buccotomy was performed after extraction if needed. The alveolus was sealed with polyvinylsiloxane (Zhermack, Rovigo, Italy) to avoid entry and packing of food for 14–21 days.

Relevant information about the cases was retrieved from the medical records and recorded, including case details for each patient, diagnostics performed, affected tooth, intraoperative difficulties, tooth size, surgical outcome, procedure duration, post-operatory complications, and medication and hospitalization time. An extraction was considered successful when a tooth was removed from the alveolus without the need for another technique. In cases of failure, the technique used to accomplish extraction was also recorded. Follow-up of all clinical cases was performed via a telephone conversation with owners or referring veterinarians one year after tooth extraction. 

## 3. Results

### 3.1. Cadaver Study

**Figure 4:**
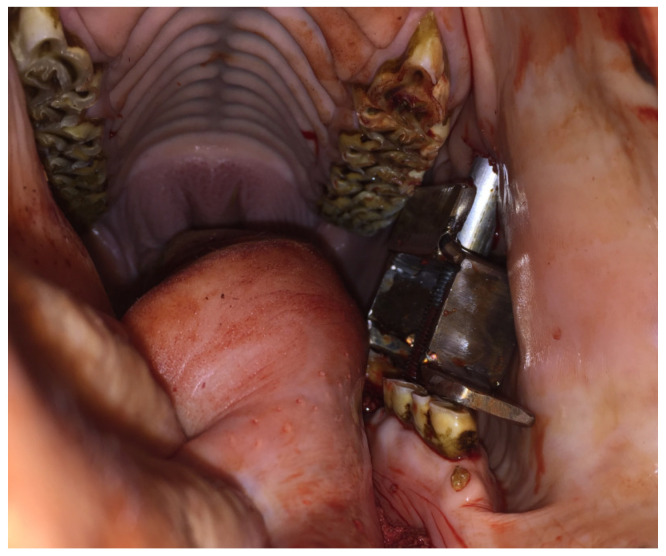
Image of the CSET device being used to extract a 307 tooth in one of the cadaver’s heads. Drilling the tooth longitudinally was difficult, and the crown fractured during the extraction.

Sixteen cheek teeth (ten superior and six inferior) underwent attempted extraction in eight heads, two teeth in each head (Table 1). CSET was successful, defined as intact tooth removal in 12 out of the 16 procedures (eight superior and four inferior teeth). The technique failed in three procedures due to crown fracture. This complication occurred in two cases because drilling was not performed longitudinally on the tooth and, in one case, because the bridge was not correctly placed on the adjacent teeth. In one case, the placement of the bridge on a 210 was not possible because of the lack of space. Superficial damage to the oral mucosa was observed in 12 out of the 16 extraction attempts. This was caused by direct pressure of the device against the cheek. Drilling longitudinally to the tooth was found to be more complicated on inferior teeth compared to superior teeth (Figure 4).

### 3.2. Retrospective Clinical Report

Corkscrew dental extraction was attempted in 24 clinical cases between October 2016 and March 2021 (Table 2). Patients included 16 males (both geldings and stallions) and eight mares, with a mean age of 8.6 ± 5.11 years old (median 7.5 years old) at the time of dental extraction. There were 10 Andalusian horses (PRE), eight crossbred horses, three Spanish sport horses, one Hannoverian, one Lusitano, and one mule. 

Oral examination and radiographs were performed on all horses to evaluate the need for extraction. In one horse, computed tomography was also performed to confirm a concurrent maxillary bone fracture. A total of 25 cheek teeth were extracted from 24 horses. Apical infection alone (17/25) was the most common condition for extraction, but apical infection with concurrent crown fracture (1/25), chronic sinusitis (4/25), cementoma (2/25), and maxillary bone fracture (1/25) were also causes of extraction. Draining tracts prior to extraction were present in 3 out of 25 cases. Superior cheek teeth were extracted in 22 procedures, and inferior cheek teeth in 3 procedures. Most of the procedures were performed in the superior Triadan 07. One horse had two adjacent cheek teeth extracted. 

Twenty-four out of 25 CSETs were performed in the standing sedated animal. One animal did not tolerate oral manipulation with the Günther speculum in place, and general anesthesia was required to complete the extraction. 

There were 14 intraoperative complications recorded in 12 procedures. Fractures of the tooth crown (2/14) and thread stripping (4/14) were the major complications that led to the failure of the CSET. Partial crown fracture (2/12) did not compromise extraction CSET. Small root fragments were observed in radiographs after three successful corkscrew procedures and had to be extracted with elevators and forceps through the buccotomy. Profuse hemorrhage through the buccotomy was seen in one procedure due to the perforation of a branch of the facial artery. The average length of the extracted teeth was 7.2 ± 1.20 cm (range from 4 to 9 cm). The tooth length challenged its removal from the arcade in 2/14 cases.

The CSET was successful in 19/25 extraction procedures and unsuccessful in 6 procedures (four superior and two inferior). For unsuccessful superior cases, extraction was completed by fracturing the remaining tooth and removing the fragments with the help of instruments placed through the buccotomy. Repulsion was only used to complete the extraction of unsuccessful mandibular cases. The average extraction time for all procedures was 108 ± 57 min (range 45–240 min). Some amount of purulent discharge of the buccotomy was observed in all cases, but it resolved within 5–10 days after surgery without any specific treatment. The mean hospitalization time after extraction was 5.8 ± 6.89 days (range 1–31 days). 

Follow-up was available for 22 out of 24 horses one year after tooth extraction. One horse died from colic 8 months after tooth extraction and one horse was sold abroad. All 22 horses available for follow-up were reported to have healed fine after tooth extraction and had no complications or sequela with the affected alveolus or the adjacent teeth. 

## 4. Discussion

This study provides clinical evidence that the CSET is an effective method to extract cheek teeth in horses under standing sedation without the use of a mallet. All cheek teeth positions, except for Triadan 11s, were attempted in our study either in cadaver heads or clinical cases. Triadan 11s were excluded from the study because their reserve crown is curved in a rostral direction [16] making it impossible to drill the tooth longitudinally. Also, there is no caudal support for the corkscrew mechanism. Their caudal position dictates that even with the mouth fully opened and lateralized, there is no space between the occlusal surfaces of the superior and inferior cheek teeth. Of the attempted tooth positions, the CSET was feasible in all except for the inferior Triadan 10s. We found it very complicated, if not impossible, to longitudinally drill the Triadan 410 due to its caudal position and because the mandible is narrower than the maxilla. Similar difficulties were found in drilling the second superior molars because of their caudal position, but the wideness of the maxillary bone allowed better access for the drill and mechanism placement. These challenges have been reported by other authors using the screw extraction technique [7,13,14].

An essential part of our dental extraction technique is the placement of an intraoral bridge to apply the extraction force of the corkscrew mechanism, which means that we always need rostral and caudal support for the bridge on the teeth adjacent to the targeted one. However, when approaching the Triadan 06, we had no rostral support, so we had to overcome this problem by placing a small plastic block under the rostral edge of the bridge to push against the maxillary/mandibular bone. Apart from mild mucosal damage, we did not find any long-term problems in the clinical cases due to the pressure applied on the gingiva and bone with the block. Additionally, in cases in which an adjacent tooth to the targeted one was missing, a wider bridge was used to obtain support from the next nearest tooth.

The main purpose of developing our technique was to avoid the use of the mallet on the heads of the horses. In our experience with the transbuccal screw extraction technique [13,14], the mallet is not very well tolerated by some horses, especially in young animals with long teeth and intact periodontal ligament. In these cases, the mallet must be used repetitively and vigorously to extract the tooth, which can make the procedure dangerous for the horse and the personnel involved in the surgery. Another problem related to the use of the mallet is a fracture of the tooth because an uneven force is applied at each strike. In our clinical cases, 24 out of 25 extractions were completed standing with sedation and nerve blocks. Most of the horses tolerated the CSET very well. Only one horse had to be anesthetized because it did not allow his mouth to be opened with the speculum, making the procedure dangerous. 

The success rate of the present technique is 76%, with failure being recorded in 6 out of 25 procedures (24%). The overall success rate of CSET is slightly lower than with the intradental screw extraction technique [14] and lower than the oral extraction technique [4]. This can be the result of the low number of cases in our study and us being at the beginning of the learning curve for this technique. Gingiva separation and pre-intraoral loosening of the targeted teeth were not performed in our study, but they could facilitate corkscrew extraction. This is considered an important step to maximize the outcome of the screw extraction techniques [14] and could contribute to raising the success rates in future procedures. Additionally, we believe our instruments need to be refined by a more specialized blacksmith so the technique can be improved.

CSET failure occurs mainly for two reasons: either because of misaligned drilling or thread striping. A crown fracture can be linked with misalignment of the drilling bit in relation to the tooth axis. When the drill hole is not longitudinally aligned with the tooth crown, the corkscrew system will apply an oblique force (lever-like action) on the crown. As the hard tooth’s crown is not very plastic, it cannot adapt to this oblique force and breaks along the hole. Thread stripping is associated with small drill holes and misalignment during thread cutting. We found that CSET fails more often in smaller teeth. The smaller the teeth, the smaller the drilling hole and the lesser the tooth substance to be purchased by the screw, making it more prone to thread stripping. Furthermore, we observed that our tap could not cut threads in the entire length of the hole, meaning that in smaller teeth, only a few screw threads could be cut. 

We believe that our technique could perform better in non-ideal conditions, such as lack of tooth substance and friable material because the force exerted by nut tightening is more uniform and gentler when applied than in the case of mallet strikes. We do not find that partial clinical crown fracture dictates failure of the technique, as in most of the procedures where this complication was present, the tooth was successfully removed. We think that even with a fracture of the clinical crown, enough intact tooth substance is present in an apical direction that it would permit appropriate screw placement. When the corkscrew fails, the cannula permits access to straight instrumentation that facilitates the use of other extraction methods. When CSTE fails due to tooth fracture at the screw hole, the tooth usually becomes small fragments that can be elevated and extracted individually using long forceps through the buccotomy.

Reported complications related to the buccotomy approach include iatrogenic damage to the parotid duct, to the facial artery and vein, and to the branches of the facial nerve [13,14,17]. Facial nerve paralysis, wound swelling, and cheek hemorrhage are also reported [13,14,17]. In this study, profuse hemorrhage through the buccotomy site was seen in one procedure only. The bleeding was controlled after ligation of a small buccal artery. Some purulent discharge from the buccotomy site was observed in all clinical cases, but healing still occurred within 7–10 days after surgery. In our study, the buccotomy site was left open to heal by the second intention. Langeneckert et al. (2015) closed primarily the buccotomy and reported 7% of complications. Very careful identification of the anatomic structures, the precise location of the buccal incision, and the use of a trocar sleeve to protect the tissues from the instruments may be the reasons for a low incidence of complications with buccotomy [14,17].

Oral extraction can be performed by any equine veterinarian with good technique, knowledge, careful case selection, and good extraction planning with other alternative methods. The grade of attachment of the targeted tooth in the alveolus, the integrity of the clinical crown, and the root morphology are important factors for a successful oral extraction. This extraction method relies on the use of various specialized instruments and several ranges of movements to disrupt the periodontal ligament. It can be a time-consuming and frustrating procedure [1,6]. Repulsion techniques and alveolar osteotomy are the most widely accepted alternatives when oral extraction fails [5,6,7,10], although they have higher rates of complications as slow alveolar healing, oroantral fistula formation, and damage needing repulsion of an adjacent cheek tooth can occur [4,8,12]. Alveolar sequestrum occurred after two procedures and complete alveolar healing occurred after the necrotic bone fragment had been removed. Extraction techniques involving the use of an intradental screw are reported to cause less damage to the anatomical structures adjacent to the cheek teeth [14,17].

As anticipated, surgery time was shorter in case of successful extraction with the corkscrew. When the first technique fails, and another technique is used, there is an expected increase in the procedure duration. The duration of CSET procedures is similar and, in some cases, faster than the duration of oral techniques, which could last more than one hour [1]. 

## 5. Conclusions

In conclusion, CSET can be safely performed in standing sedated horses, and this technique avoids the use of a mallet. Very precise alignment of the drill bit with the longitudinal axis of the tooth is the most important step for successful extraction. The technique is performed more easily in the superior Triadan, as the anatomy facilitates the drilling angulation. Bridge placement is more difficult in the second molars due to reduced space at this level. As with other minimally invasive buccotomy and intraoral screw techniques, some complications are expected to occur with CSET. Further research on postoperative long-term complications needs to be performed on the presented technique in order to make it a true alternative to the current surgical approaches and screw extraction techniques. The use of a right-angle drill and a smaller “corkscrew” system could exclude the need for buccotomy and may permit an approach to the more caudal cheek teeth.

## Figures and Tables

**Table 1:** CSET cadaver study details (modified Triadan positions).

Head	Tooth	Outcome	Difficulties
1	107	Success	Not observed
	207	Failure	Crown fracture
2	106	Success	Not observed
	208	Success	Not observed
3	306	Success	Not observed
	307	Failure	Drilling angulation + crown fracture
4	209	Success	Not observed
	408	Success	Drilling angulation
5	110	Success	Bridge placement
	309	Success	Not observed
6	210	Failure	Bridge placement
	406	Success	Not observed
7	108	Success	Not observed
	109	Success	Not observed
8	208	Success	Not observed
	410	Failure	Drilling angulation + crown fracture

**Table 2:** Clinical case details (modified Triadan positions). M—male; F—female; PRE—Andalusian horse; X—crossbred; CDE—Spanish sport horse; Hann—Hannoverian; PSL—Lusitano; AI—Apical infection; Fx—tooth crown fracture; CS—chronic sinusitis; CF—cutaneous fistula; MBFx—maxillary bone fracture; CMT—cementoma; N/O—not observed; PFx—partial crown fracture; TS—thread stripping; RFx—tooth root fracture; HM—buccotomy hemorrhage; S—successful; U—unsuccessful; AS—alveolar sequestrum; BI—buccotomy infection.

Case	Age (Years)	Sex	Breed	Diagnosis	Affected Tooth	Tooth Size (cm)	Intraoperative Difficulties	Surgical Outcome	Surgery Duration (Hours)	Postoperative Complications	Hospitalization Time (Days)
1	10	M	PRE	AI	106/107	6.7/6.9	N/O	S	2.00	N/O	7
2	11	M	X	AI	107	6.5	Fx	U	3.50	N/O	5
3	3	M	PRE	AI + CF	108	9.0	SIZE	S	2.00	N/O	5
4	4	F	PRE	AI	109	7.4	PFx	S	2.50	N/O	2
5	10	M	PRE	AI	207	7.0	N/O	S	1.15	N/O	2
6	3	M	CDE	AI	207	8.0	TS	U	3.00	N/O	4
7	4	M	CDE	AI	208	9.0	N/O	S	1.00	N/O	4
8	14	M	X	AI	208	6.0	N/O	S	1.50	N/O	3
9	8	F	X	AI	209	6.5	PFx + RFx	S	1.00	N/O	3
10	7	M	Mule	AI	209	8.0	TS	U	3.00	N/O	5
11	4	M	X	AI	107	9.0	N/O	S	1.00	N/O	1
12	16	F	CDE	AI + Fx	409	5.0	TS	U	2.50	N/O	1
13	14	F	X	AI + CS	210	6.5	RFx	S	1.50	AS	31
14	6	F	Hann	AI + CF	107	8.5	SIZE	S	0.75	N/O	3
15	3	M	PRE	AI	109	9.0	N/O	S	1.50	N/O	1
16	4	M	PSL	AI + MBFx	107	7.0	N/O	S	1.25	AS	16
17	5	M	PRE	AI + CMT	107	8.0	N/O	S	0.75	N/O	1
18	20	M	X	AI + CS	209	4.0	HM + TS	U	4.00	BI	15
19	12	F	PRE	AI + CF	308	6.0	Fx	U	3.00	N/O	5
20	5	M	PRE	AI	106	7.0	N/O	S	0.75	N/O	2
21	10	M	PRE	AI + CMT	208	7.5	N/O	S	1.25	N/O	3
22	4	F	PRE	AI	307	7.0	N/O	S	1.50	N/O	0
23	18	M	X	AI + CS	209	6.6	N/O	S	1.25	N/O	15
24	12	F	X	AI + CS	109	7.2	RFx	S	1.00	N/O	7

## Data Availability

The data presented in this study are available upon request from the corresponding author.
